# The Susceptibility of Convalescents from Measles to Streptococcus Infections1.For a complete account, see article by R. L. Levy and H. L. Alexander, *Journal of the A. M. A.*, July 15, 1918, p. 1827.

**Published:** 1918-11

**Authors:** Robert L. Levy


**The Susceptibility of Convalescents from Measles to Streptococcus Infections**[1]. Captain Robert L. Levy, M. C.

The speaker said in part :

During the past winter and spring, it became evident, from reports coming from various army cantonments in the United States, that measles, because of the severity and frequency of its complications, must be regarded as a serious camp disease. Complications necessitate long hospitalization and cause, therefore, a high non-effective rate; bronchopneumonia and empyema were responsible for many deaths. With surprising uniformity, the complications were due to infection with a hemolytic streptococcus.

In the report of a Commission sent by the Surgeon-General of the Army to study the pneumonia at San Antonio, Texas, two significant facts concerning the relation of the streptococcus to the complication of measles were pointed out : first, a relatively small number of measles patients (11.4 per cent.) harbored this organism in their throats on admission to the hospital; second, many more acquired S. haemolyticus in their throats for the first time during their stay in the wards. It was therefore inferred that at fort Sam Houston, at least, the high incidence of bronchopneumonia and empyema during convalescence from measles might be directly related to the transfer of the infectious agent from one patient to another in the wards of the hospital.

An epidemic of measles at Camp Zachary Taylor, Kentucky, afforded an unusually good opportunity for extending these observations. 388 cases, on which careful clinical records were kept and full bacteriological studies made, form the basis of this report.

### Plan of the Present Study.

*Ward Routine.* On admission to the hospital all cases of measles were sent to a special " measles receiving ward ", which was divided into cubicles by sheets, and where every patient was

1. For a complete account, see article by R. L. Levy and H. L. Alexander, *Journal o the A. M. A.*, July 15, 1918, p. 1827.

required to remain in bed. Each day throat cultures were taken from those admitted during the preceding twenty-fours hours. Carriers of S. haemolyticus were on the following morning sent to the " dirty " wards. Those whose cultures were negative for this organism were held for another day, and, if a second swabbing confirmed the finding of the first, were sent to the " clean " wards. Rarely did the results of the second culture contradict the report of the preceding day. All patients in each ward were swabbed again at least once a week.

A separate ward was set aside for the reception of post-measles pneumonia, to which, immediately upon the recognition of this complication, patients were transferred.

In both " clean " and " dirty " wards, cubicle isolation was strictly observed. Each patient was provided with a gauze mask, changed every twenty-four hours, and was required to wear this mask constantly when not in his own cubicle. For infringements of this rule, punishments were meted out by the ward surgeon, who also daily gave a short talk to ward personnel and patients on the rationale of the various measures for isolation. In this way, better co-operation was insured. War surgeons, nurses, and orderlies, when in the ward, were properly masked and gowned.

All patients, in bed or convalescent, were fed in their own cubicles; three or more waiters, chosen from among the convalescents, served the food. At all times, but especially during meal hours, crowding together was prohibited; each man ate either in bed or at his own bedside. In the washroom, only one man was admitted at a time; in the latrines, the masks were worn and no more were allowed to enter than could be accommodated at any one time. A guard, changed every two hours and chosen from among the convalescents, was stationed at the door of the latrines to insure the enforcing of these rules.

It may well be asked why such rigid precautions were observed in wards in which only carriers or non-carriers were cared for, and why these measures are described in detail. As pointed out by Cole, and repeatedly confirmed in our own experience, S. haemolyticus is very readily transferred from one individual to another, and, no matter how strict the supervision, an occasional lapse in discipline is bound to occur. The slightest break in the chain of precautions may result in disaster. For example, in a " clean " ward, throat cultures made at intervals of a few days revealed the fact that several " clean " cases were being converted into carriers. On swabbing the personnel of the ward, it was found that two orderlies and a nurse harbored S. haemolyticus in profusion in their throats. On replacing these individuals with non-carriers, no further contamination occurred. This experience taught us to provide the clean wards with a personnel of non-carriers. In the " dirty " wards, it is desirable to prevent one individual from becoming a carrier of another patient's strain of streptococcus, for different strains may vary in virulence.

*Laboratory Technic* : Cultures from the pharynx and tonsils were taken at the bedside with straight swabs, which were then placed into about 1 cc. of beef infusion broth, shaken and brought to the laboratory. There they were plated, without incubation, on 10 o/o human blood agar plates. In order to insure proper separation of colonies, it was essential to rinse each swab thoroughly by twirling it forcibly against the side of the test tube, then wiping it further at the periphery of the plate and streaking lightly. After 18 or 20 hours incubation, single hemolytic colonies suggestive of streptococcus were transplanted into beef infusion broth and grown for 24 hours, when cultural characteristics, staining reactions, and morphology were noted. Bile solubility was done only when pneumococcus was suspected. Fermentation reactions and degree of hemolysis were not studied.

Twenty sputum cultures were made by spreading a fragment of thoroughly washed sputum over the surface of a blood agar plate. Bronchial secretions were obtained when possible. Of thirteen cultures from streptococcus carriers, and seven from non-carriers, all corresponded to the throat swab findings, thus demonstrating that the throat culture is apparently an index of the presence or absence of S. haemolyticus in the lower respiratory tract.

*Incidence of Streptococcus Carriers in Measles Patients Admitted to the Hospital.*

The following table is self-explanatory :

TABLE I

*Results of Throat Cultures in Measles Admissions.*

| | Number. | Percent. |
|---|---|---|
| Non-carriers. . . . . . . . . . | 89 | 22.9 |
| Carriers. . . . . . . . . . . . . | 299 | 77.1 |
| Total number of cases . . | 388 | 100.0 |

These figures are in striking contrast to the percentages prevailing at Fort Sam Houston, Texas, where only 11.4 o/o of measles patients were carriers of S. haemolyticus on admission to the hospital.

Of the 89 non-carriers admitted to the wards, 27 became infected during the period of their hospitalization. Contamination for the most part occurred during the early stages of the work, when the ward routine had not been thoroughly established. These cases are, of course, classed with the carriers in the final analysis.

*Relative Incidence of Complications Among Carriers and Non-Carriers*

Coryza, bronchitis, and a laryngitis of variable severity, associated with an initial fever of from three to six days duration, were almost constant early accompaniments of each case. A majority of patients were admitted on the second or third day of the disease, and fever occurring after the tenth day of hospitalisation was regarded as pathological. Of the 388 cases, 119, or 30 o/o, had complications, a number of patients suffering from more than one. The complications are charted in the accompanying table in order of frequency.

TABLE II

*Complications.*

| Complications. | Carriers. | Non-Carriers. |
|---|---|---|
| Bronchopneumonia . . . . . . . . . | 47 | 0 |
| Acute Tonsilitis. . . . . . . . . . . | 25 | 1 |
| Acute Bronchitis. . . . . . . . . . | 23 | 2 |
| Acute Suppurative Otitis Media . . . | 22 | 0 |
| Empyema . . . . . . . . . . . . . | 15 | 0 |
| Acute Sinusitis. . . . . . . . . . . | 10 | 0 |
| Peritonsillar Abcess. . . . . . . . . | 7 | 0 |
| Erysipelas . . . . . . . . . . . . . | 6 | 0 |
| Cervical Adenitis. . . . . . . . . . | 5 | 1 |
| Peritonitis . . . . . . . . . . . . . | 1 | 0 |
| Septic Meningitis . . . . . . . . . | 1 | 0 |
| Total . . . . . | 162 | 4 |

An analysis of this table brings out a number of interesting points :

1. The complications of measles occurred almost exclusively among streptococcus carriers, the incidence in this group being 36.8 o/o as contrasted with 6.4 o/o in " clean " cases. Furthermore, the four complications noted among the non-carriers were of distinctly minor nature, — two instances of acute bronchitis, one acute tonsillitis, and one cervical adenitis.

2. Bronchopneumonia occurred 47 times, or in 12.1 o/o of all cases. This complication was responsible for many deaths; accurate mortality statistics were not available at the time of the present writing, but about one half of the cases died. Fifteen, or 34 o/o, of the bronchopneumonias developed empyema.

3. On culture, 13 pleural fluids showed S. haemolyticus, one pneumococcus. From 11 specimens of pus obtained from cases of acute otitis media, S. haemolyticus was grown 9 times; staphylococcus aureus twice.

4. Of 326 carriers, either entering the hospital as such or becoming infected after admission, 211, or 63.2 o/o, had no complications.

*Acquisition of the Carrier State in the Wards.*

Reference has already been made to Cole's observations on the case with which S. haemolyticus may be spread from one individual to another in the ward of a hospital. Experiments were carried out in which wards were " mixed ", that is, were filled half with " clean ", half with " dirty " cases, in alternate beds. Newly admitted cases of measles, convalescents, and patients with complications composed the ward roster. All the precautions previously noted were rigidly enforced in order to prevent the contamination of " clean " by " dirty " cases. Yet, in one ward in which 15 " clean " and 15 " dirty " cases were placed, at the end of a week only 6 non-carriers remained. In another ward of 24, of whom at the start 12 were non-carriers, at the end of the week only 3 " clean " cases were found. These observations emphasize the importance of rigidly observing every precaution to prevent contact spread, though casting considerable doubt upon the efficacy of the methods herein described. Therefore, when possible, if the incidence of complications is to be reduced, carriers and non-carriers should be separated, and cared for in different wards. This procedure should prove particularly valuable in those camps where the number of streptococcus carriers among measles patients is low, as at Fort Sam Houston, Texas.

*Difficulties of Eradicating the Carrier State.*

Throat cultures made at intervals in many of the " dirty " wards showed that the carrier state, once acquired, persists throughout the patient's stay in the hospital. There were rare exceptions to this rule.

Attempts at mouth disinfection were not successful. Dakin's solution, in half strength, in common use as a gargle and spray in many army hospitals, will not kill S. haemolyticus, even *in vitro*. Experiments with other mouth antiseptics, notably iodine in gly cerine, though successful in the test tube, were clinically most disappointing. The organisms, apparently safely lodged in the crypts of the tonsils and in the lymphatic tissues of the nasopharynx, were not accessible to the local treatments so far employed. 71.7 o/o of the patients discharged from the hospital still harbored S. haemolyticus in their throats.

*Epidemiology.*

It was shown earlier in the year that Camp Zachary Taylor was heavily infected with S. haemolyticus, and during the winter representatives from almost every organization entered the Base Hospital with lesions caused by this organism. In the present epidemic of measles, it was striking that of the 388 cases studied, 346, or 89.1 o/o, came from the 159th Depot Brigade, an organization into which all new men entering camp were attached during the early period of their training. Accordingly, throat cultures were made on 95 men of Company 5 (2nd Battalion), composed of individuals from various parts of the camp, most of them having been in the service about six months. Of these 70, or 83.2 o/o, were carriers of S. haemolyticus, 64.3 o/o being heavily infected, 18.9 o/o showing only an occasional colony on the plates. All were apparently healthy.

If these observations made on a representative company may be used as an index, a high percentage of soldiers in the Depot Brigade harbored S. haemolyticus in their throats. The question naturally arose, did these men come from civil life as carriers, or did they acquire the organism in the camp? A recent draft afforded an exceptional opportunity for determining this point. Throat swabs were taken from 489 new recruits, representatives of both urban and rural communities, as they stepped from the train. Of these cultures, 14.8 o/o showed the presence of hemolytic organisms.

The inference is obvious. It was previously shown that Camp Zachary Taylor was heavily seeded with S. haemolyticus. A large number of the men, therefore, acquired the streptococcus carrier state during their sojourn in camp.

In conclusion, it may not be amiss to call attention to the analogy which exists between measles and the affection, which, for want of a better name, is termed " influenza ". Both render the upper respiratory passages peculiarly susceptible to invasion by secondarily invading micro-organisms; in both, pneumonia and empyema of a particularly virulent variety are serious and highly fatal complications.

In the prophylaxis and treatment of cases of influenza, which at present are all too numerous in the Allied Armies, particularly in the A. E. F., it would seem desirable, therefore, to take even more rigid precautions than heretofore to prevent the spread of this infection and to reduce to a minimum the danger of transmission, from one individual to another in hospital wards, of those bacteria responsible for pneumonia, empyema, and other complications.

